# Influence of Steel Structure on Machinability by Abrasive Water Jet

**DOI:** 10.3390/ma13194424

**Published:** 2020-10-05

**Authors:** Irena M. Hlaváčová, Marek Sadílek, Petra Váňová, Štefan Szumilo, Martin Tyč

**Affiliations:** 1 Department of Physics, Faculty of Electrical Engineering and Computer Science, VSB-Technical University of Ostrava, 708 00 Ostrava, Czech Republic; martin.tyc.st@vsb.cz; 2 Department of Machining, Assembly and Engineering Metrology, Faculty of Mechanical Engineering, VSB-Technical University of Ostrava, 708 00 Ostrava, Czech Republic; marek.sadilek@vsb.cz; 3 Department of Material Engineering, Faculty of Materials Science and Technology, VSB-Technical University of Ostrava, 708 00 Ostrava, Czech Republic; petra.vanova@vsb.cz (P.V.); stefanszum@gmail.com (Š.S.)

**Keywords:** abrasive water jet cutting, surface roughness, heat treatment, hardness, tensile strength

## Abstract

Although the abrasive waterjet (AWJ) has been widely used for steel cutting for decades and there are hundreds of research papers or even books dealing with this technology, relatively little is known about the relation between the steel microstructure and the AWJ cutting efficiency. The steel microstructure can be significantly affected by heat treatment. Three different steel grades, carbon steel C45, micro-alloyed steel 37MnSi5 and low-alloy steel 30CrV9, were subjected to four different types of heat treatment: normalization annealing, soft annealing, quenching and quenching followed by tempering. Then, they were cut by an abrasive water jet, while identical cutting parameters were applied. The relations between the mechanical characteristics of heat-treated steels and the surface roughness parameters *Ra*, *Rz* and *RSm* were studied. A comparison of changes in the surface roughness parameters and Young modulus variation led to the conclusion that the modulus was not significantly responsible for the surface roughness. The changes of *RSm* did not prove any correlation to either the mechanical characteristics or the visible microstructure dimensions. The homogeneity of the steel microstructure appeared to be the most important factor for the cutting quality; the higher the difference in the hardness of the structural components in the inhomogeneous microstructure was, the higher were the roughness values. A more complex measurement and critical evaluation of the declination angle measurement compared to the surface roughness measurement are planned in future research.

## 1. Introduction

The technology of Abrasive waterjet (AWJ) has been widely used in many areas of human activity for several decades. The physical principle of the process is the transfer of mechanical energy from the pump to material and its use for required operations, namely various types of material disintegration such as milling [1,2], machining of composites [3], rock breaking [4], wood cutting [5], and many others. One of the most frequent and important applications of this technology is material cutting. Its efficiency should be evaluated from two points of view: either by the amount of consumed energy along with the costs, or by the evaluation of the quality of the cut, namely the surface roughness and cutting accuracy [2,5,6,7,8]. In the case of plain dividing cuts, the quality is usually not the most important thing. However, it may become the key problem in complex engineering processes.

It is rational to suppose that the quality of the cut would be influenced by material characteristics. The father of the AWJ technology, Hashish, originally identified material strength as an important characteristic influencing the AWJ cutting process [9]. However, in his erosion model for predicting the depth of the cut of abrasive waterjets in different metals [10], he described the correlation with the elastic modulus and dynamic flow stress, one of the two main properties he needed for a metal cutting process characterization. Later on, Zeng and Kim studied the correlation of various material parameters: the material structure element size, flaw distribution, Young’s modulus of elasticity, Poisson’s ratio, fracture energy, density, flow stress, AWJ cutting efficiency and the quality of the surface [11]. In a model presented by Che et al. [12], the influence of the material hardness on the surface roughness was investigated. Vikram and Babu developed a model for the numerical simulation of the cutting process, introducing material density [13]; Deam et al. [14] based a theoretical 2D model on the cutting wear factor introduced by Bitter [15,16]. There has been a lot of other interesting research work since then [17]. Whereas in models published in [11,12,13,14], the data used for the proposed models’ verification came from the cutting of nonmetal, rather brittle, materials, e.g., granite, perspex or ceramics, other authors focused their attention on metals, like Hashish did. Arola and Ramulu found that the depth of subsurface plastic deformation is inversely proportional to a metal’s strength coefficient [18], Chen et al. improved the cutting quality of mild steel by controlled nozzle oscillations [19], Hascalik et al. studied the depth of cut and smooth cutting region on titanium [20], Hlaváč et al. proposed a method for the compensation of the jet retardation and the taper [21], and Monno et al. studied the influence of heat treatment on the kerf roughness of carbon steel cut by AWJ [22]. Some of these researchers prepared models for the process description, while others focused on specific problems, among which the geometric accuracy, surface integrity and kerf roughness are achieving much attention [22,23,24]. Roughness might be important not only for manufacturers but also for material engineers, due to its potential to reduce necessary final polishing in cases when AWJ is used for cold cutting in sample preparation [25]. Although a lot of research was done in the search for a direct and simple connection between the material parameters and the cutting quality, and some unsubstantiated theories were published, no reliable proofs of such a linkage has been brought forward up to now.

One of the most interesting and inspiring works is the research of Strnadel et al. [26], who studied the relationships between the declination angle introduced by Hlaváč [27] and selected material properties, namely the strength, hardness and material element size. Although their research was aimed at another problem, they announced that their results indicated the dependence of the AWJ declination angle not only on strength characteristics, but also on the microstructure of the steel—i.e., on the way in which plastic deformation occurred during the AWJ–material interaction. Mono et al. [22] tried to trace this dependence in more detail and studied carbon steel C40 subjected to two different heat treatments and cut with different traverse speeds. They found that workpiece hardness affected the surface finish in different ways depending on the AWJ cutting parameters.

Our research group investigated the changes in the interaction of AWJ and steels with different microstructures under standard cutting parameters usual for the chosen thickness of the steel material. Three different steel grades suitable for heat treatment were chosen, and experiments aiming to reach significant changes in the steel microstructure were designed.

Carbon steel C45 represents a structural carbon steel for quenching and tempering, surface hardening, and for smaller and large forgings. It is suitable for shafts of mining machines, turbochargers, compressor pistons, etc.; its weldability is very difficult [28].

Steel 37MnSi5 is a manganese-silicon steel for tempering, and for large and larger forgings. It is relatively easy to form and also easy to machine. It is very susceptible to tempering brittleness. The steel is used for medium-stress machine and motor vehicle components and is particularly wear-resistant. The optimal diameter for finishing is 50 mm; its weldability is also very difficult [29].

Steel 30CrV9 represents a low-alloy steel with good weldability and machinability, which is suitable for finishing, surface quenching, and chemical-thermal and nitriding treatment. It is used on heavily stressed hardened machine parts and on nitrided parts, including nitrided gears. Due to its high hardenability, it can be used for large forgings. It is also used for the crankshafts of aircraft engines, propeller heads, connecting rods, connecting rod bolts and similarly stressed machine parts like the drive axles of motor vehicles, steering levers, etc. [30].

Our research was aimed at testing the validity of the findings presented in [26] for other surface quality indicators than the declination angle, namely *Ra* and *Rz*, which are more generally applied in engineering. We also included the mean width of the assessed profile *RSm* into our surface roughness evaluation, as we supposed it to be potentially related to the internal structure of the material.

## 2. Materials and Methods

This paper deals with the influence of the microstructure of steels having passed through various types of heat treatment on the cutting properties of an abrasive water jet. For this experiment, three different types of steel, carbon steel C45 (W.Nr. 1.0503 or 1.1191 equivalent), micro-alloyed steel 37MnSi5 (W.Nr. 1.5122 equivalent) and low-alloy steel 30CrV9 (W.Nr. 1.7361 equivalent), were used. The materials were chosen so as to maximize the changes in their characteristics induced by heat treatment. As the amount of carbon was different in each of them, different changes in the steel microstructure could be expected. The chemical composition of steels determined by glow discharge optical emission spectrometry (GDOES) [31] on a GDA 750 instrument, produced and supplied by Spectruma analytic Gmbh, is given in Table 1.

Originally, there were hot rolled round bars with a diameter of 200 mm. Discs 30 mm thick were cut from these bars and divided into quarters. Each quarter was subjected to a different heat treatment, namely: normalization annealing, soft annealing, quenching and quenching followed by high temperature tempering. The parameters recommended by the producer (the first three columns) and the real parameters used for the heat treatment are summarized in Table 2. All three quarters of different steels were placed in the furnace and processed simultaneously in order to save time and energy. Because the recommended ranges for heat treatment are different for the three grades of steel, the efficiency of the treatment was not the same.

Both normalization and soft annealing lasted 4 h, and it was followed by cooling down either in the air (normalization) or in the furnace (soft). The quenching lasted 1 h in both cases and was followed afterwards by either cooling in water (pure quenching) or tempering (2 h in the furnace).

The change of the mechanical characteristics of the tested steels due to heat treatment was identified by a hardness measurement and tensile tests. The hardness was measured by a Vickers hardness test HV30 (under a load of 294.2 N) on transverse metallographic sections taken from the cut prisms (see below). The tensile tests were performed at room temperature on standardized test specimens [32] on the Multipurpose Dynamic and Fatigue System LFV (100 kN) produced by Walter+Bai AG, Switzerland. The loading speed was chosen to be 2 mm/min. Specimens for the tensile tests with a diameter d_0_ = 6 mm were prepared on a lathe from the prisms cut from the bulk material by AWJ.

After the heat treatment, the parts were cut with a water jet into prism-shaped test specimens with a square base measuring 10 × 10 mm and a length of 100 mm. The cutting itself was performed in the liquid jet laboratory at VŠB-TUO with AWJ generated by a high pressure pump PTV 19/60 based on HSQ FlowX5. The pump was modified by the producer in order to have adjustable pressure with a pump with adjustable pressure from 50 MPa to 415 MPa; the maximum flow was 1.9 L per min. The parameters used in the water jet cutting are shown in Table 3.

A microstructure examination of the heat-treated samples was carried out on an optical microscope Olympus GX51. The samples were grinded with 60, 80, 160, 320, 600, 800, 1200, 2400 SiC papers, then polished with a diamond suspension (crystals 1 μm) and finally etched with Nital (4% solution of HNO_3_ in ethanol) long enough to make the expected microstructures be well-distinguishable.

The surfaces cut by the above defined technology were then measured in three lines (upper line 1 mm below the upper edge, the central line, and bottom line 1 mm above the bottom edge of the sample cut surface) by a contact tester SurfTest SJ-400 (Mitutoyo) using a diamond stylus tip with radius 2 µm and cone 60°, and three required surface roughness parameters were evaluated for each line: the arithmetical mean deviation *Ra*, maximum height *Rz* and mean width *RSm*.

The arithmetical mean deviation of the assessed profile *Ra* [33] is one of the most widely used parameters. It represents the arithmetic mean of the absolute ordinate *Z(x)* within the sampling length, and it therefore provides for stable results, as the parameter is not significantly influenced by scratches, contamination and measurement noise; furthermore, it is hardly affected by individual peaks or valleys because it is the mean value of the whole profile [34]:

(1) $$Ra=\frac{1}{l}\int_{0}^{l}\left|Z\left(x\right)\right|dx$$

On the contrary, the maximum height *Rz* [33] represents the sum of the maximum peak height *Zp* and the maximum valley depth *Zv* of a profile within the reference length and so is significantly influenced by scratches, contamination, and measurement noise due to its reliance on peak values. Nevertheless, it is widely used and sometimes preferred in engineering applications [35]:

(2) Rz=Rp+Rv=max(Zp)+|min(Zv)|

The third parameter that was selected to be potentially related to the internal structure of the material was the mean width *RSm* [33]. This parameter is used to evaluate the horizontal size of parallel grooves and grains instead of the height parameters; it represents the mean value of the lengths of profile elements within the sampling length (Equation (3)):

(3) $$RSm=\frac{1}{N}\overset{N}{\underset{i=1}{\sum }}X_{si}$$

where *X_si_* means the length of the *i*-th profile element.

## 3. Results

The results are presented in three sections, introducing each of the three materials individually. Each section contains a table summarizing the measured mechanical parameters of the respective steel grade (hardness HV30 results and results of the tensile tests, i.e., lower R_eL_ and upper R_eH_ yield strength—or, if these are not significant, proof strength Rp_0.2_—ultimate tensile strength R_m_ and percentage elongation after fracture A). Another table provides the surface roughness parameters: the arithmetical mean deviation *Ra*, maximum height *Rz* and mean width *RSm*; all three lines are presented, without an average evaluation, because the interaction of the AWJ with the material traced on the bottom line is distorted by the pressure oscillations; therefore, the lines should be discussed individually.

Finally, photos of the steel microstructure after the respective heat treatments are presented and discussed. There are two photos of the same treatment; the second one with a five-time bigger magnification highlights the details of the microstructure.

### 3.1. Steel C45

#### 3.1.1. Mechanical Parameters

Various types of heat treatment induced more or less significant changes of steel mechanical parameters (Table 4). As the tensile tests of the quenched C45 steel did not reveal a clear value of lower and upper yield strength, the proof strength R_p0.2_ was evaluated.

#### 3.1.2. Microstructure

**Figure 1:**
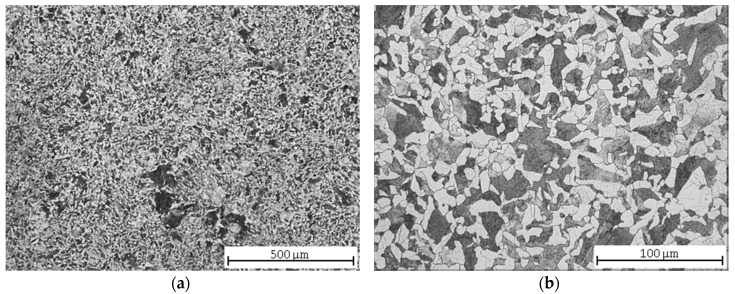
C45 steel microstructures after normalization annealing: (**a**) central part; (**b**) detail.

The microstructure of C45 steel after normalization annealing was fine-grained ferritic-pearlitic (Figure 1a) with unevenly precipitated pearlite (Figure 1b). According to [36], the steels with a lower hardness (below 270 HV) are, to a great extent, affected by other factors than hardness, namely by the pearlite nodular size. A larger pearlite size leads to a decrease of machinability in the form of an increased wear of the classic tool. Several earlier studies regarding steel machinability proved that the best machinability was provided by a spheroidized microstructure, as the amount of proeutectoid ferrite was increased in such a microstructure. The same should be expected with a smaller pearlite nodular size.

**Figure 2:**
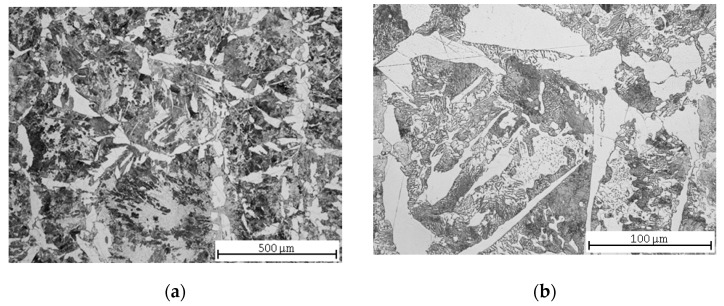
C45 steel microstructures after soft annealing: (**a**) central part; (**b**) detail.

After soft annealing, partially spheroidized pearlite and Widmannstätten morphology ferrite (Figure 2a), i.e., needle-like growths of cementite within the crystal boundaries of the martensite, became visible in the microstructure (Figure 2b). Widmanstätten structures tend to form when the coarse-grained steel is rapidly cooled, which might have occurred with the hot rolled material during production. The structure increases the brittleness of the steel, and it can only be relieved by recrystallization above A_c3_ (the final critical temperature at which free ferrite is completely transformed into austenite during heating). The soft annealing temperature of 710 °C, lying below the eutectoid temperature A_c1_ = 727 °C (the critical temperature at which pearlite transforms into austenite), notwithstanding the holding time of 4 h, is ineffective in the removal of this morphology; a complete spheroidization of cementite could not be realized; therefore, a higher brittleness of the material should be expected [37].

**Figure 3:**
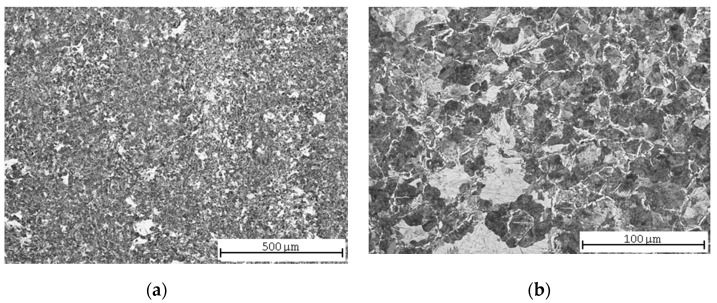
C45 steel microstructures after quenching: (**a**) central part; (**b**) detail.

As the hardenability of this steel was only 7 mm, the structure was not completely hardened. The evaluated sample might have been taken too far from the original hardened surface, and therefore the structure in Figure 3a consists mainly of pearlite and a ferritic network along the grain boundaries, while the proportion of martensite, resp. bainite is very low. The heterogeneity of the microstructure is also evident in this case (Figure 3b).

**Figure 4:**
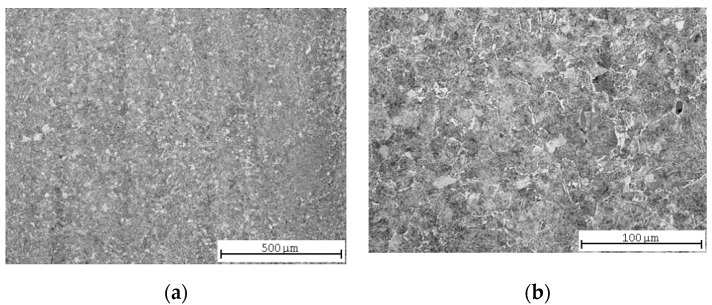
Quenched and tempered C45 steel microstructures: (**a**) central part; (**b**) detail.

After quenching and high-temperature tempering, the microstructure was formed mainly by bainite with a ferritic network along the grain boundaries (Figure 4a). The sample was taken closer to the surface in this case, and therefore the incidence of the tempered turbid microstructure was higher (Figure 4b).

#### 3.1.3. Surface Roughness Measurement

The results of surface roughness measurement provided by contact tester are summarized in Table 5. The results of the all three lines are presented, named “upper”, “central”, and “bottom” in this table. The most significant of them is the central line; the values measured on bottom lines might be distorted by AWJ pressure fluctuations.

### 3.2. Steel 37MnSi5

#### 3.2.1. Mechanical Parameters

The heat treatment of manganese steel led to different changes of mechanical parameters than it was with the carbon steel (Table 6). After quenching, this steel grade did not reveal a clear value of lower and upper yield strength, therefore, proof strength R_p0.2_ had to be evaluated both for quenched and tempered material. The changes are displayed graphically in the discussion section.

#### 3.2.2. Microstructure

**Figure 5:**
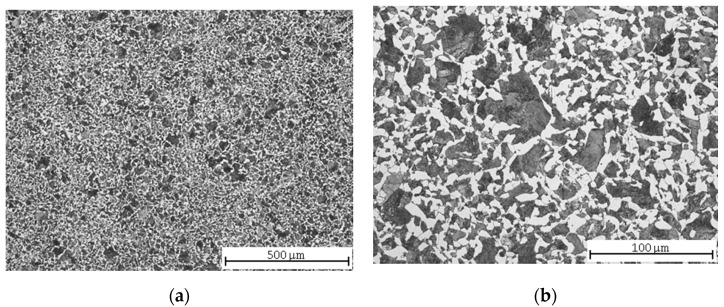
37MnSi5 steel microstructures after normalization annealing: (**a**) central part; (**b**) detail.

The microstructure of 37MnSi5 steel in the state after normalization annealing was fine-grained ferrite-pearlitic (Figure 5a) with uneven pearlite precipitation associated with low-degree annealing (Figure 5b).

**Figure 6:**
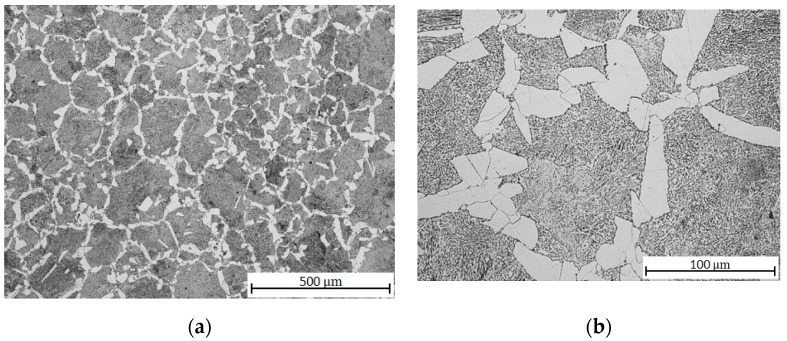
37MnSi5 steel microstructures after soft annealing: (**a**) central part; (**b**) detail.

**Figure 7:**
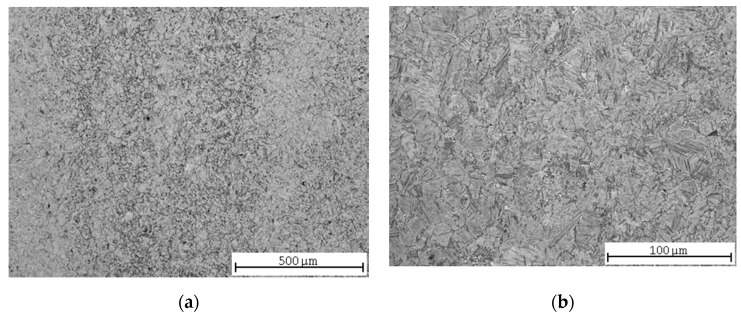
37MnSi5 steel microstructures after quenching: (**a**) central part; (**b**) detail.

**Figure 8:**
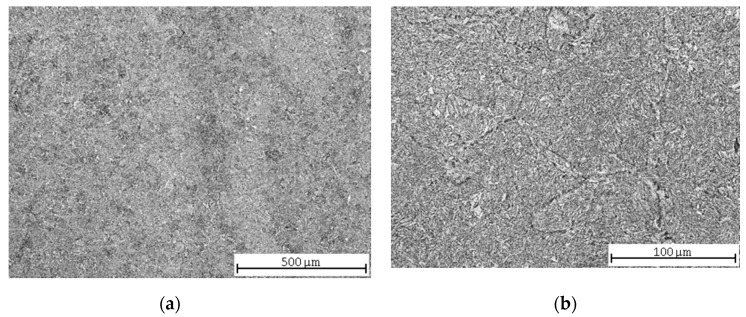
Quenched and tempered 37MnSi5 steel microstructures: (**a**) central part; (**b**) detail.

After soft annealing, an almost 100% spheroidization of the pearlite occurred (Figure 6a). Compared to C45 steel, the spheroidization rate is higher due to the lower carbon content. Ferrite is precipitated along the grain boundaries (Figure 6b). The microstructure after quenching was martensitic with a low proportion of bainite and ferrite (Figure 7a). Although the individual martensite needles are not clearly observed (they are too small), thanks to their tendency to become aligned parallel to one another in a large region of the austenite grain, their characteristic microstructure can be recognized quite well (Figure 7b). After refining, the microstructure is tempered martensitic-bainitic (Figure 8a,b).

#### 3.2.3. Surface Roughness Measurement

The Table 7 presents the results of surface roughness measurement on the three lines in similar manner as it was done for the carbon steel. They are analyzed in detail in the discussion section.

### 3.3. Steel 30CrV9

#### 3.3.1. Mechanical Parameters

The low-alloy steel 30CrV9 was the least hard steel included to this research. Its mechanical parameters after heat treatment are summarized in Table 8. Opposite to the other two steel grades it had no lower and upper yield strength, its proof strength was evaluated instead.

#### 3.3.2. Microstructure

**Figure 9:**
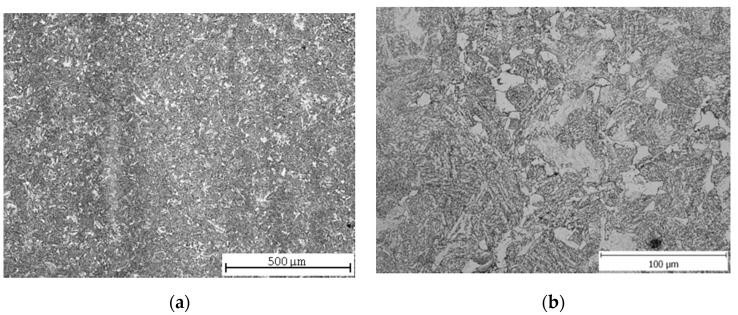
30CrV9 steel microstructures after normalization annealing: (**a**) central part; (**b**) detail.

After normalization annealing of 30CrV9 steel, the microstructure was formed by upper bainite and ferrite (Figure 9a), although the cooling was performed in air. Due to the high hardenability of this steel grade, the microstructure was partially quenched (Figure 9b).

**Figure 10:**
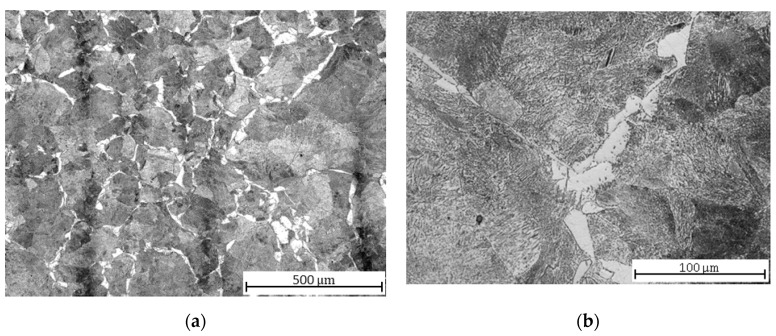
30CrV9 steel microstructures after soft annealing: (**a**) central part; (**b**) detail.

Soft annealing led to a partial spheroidization of the pearlite (Figure 10a). Due to presence of Cr and V carbides (the steel is alloyed with chromium and vanadium), spheroidization was slowed down [38]. Ferrite was precipitated along the grain boundaries (Figure 9b).

**Figure 11:**
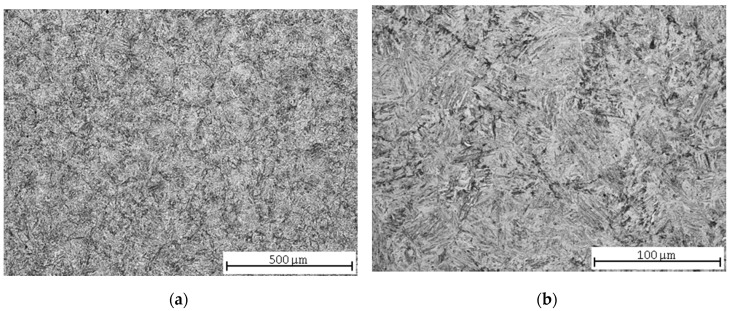
30CrV9 steel microstructures after quenching: (**a**) central part; (**b**) detail.

**Figure 12:**
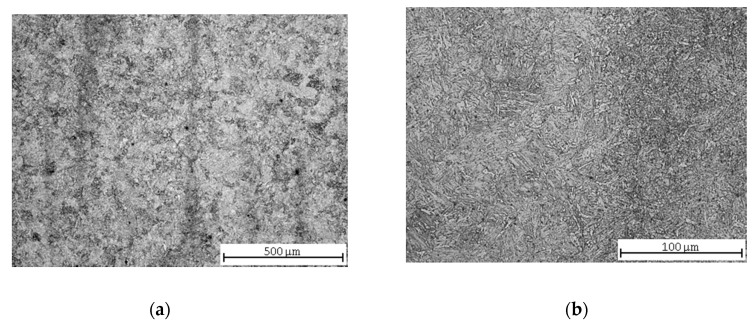
Quenched and tempered 30CrV9 steel microstructures: (**a**) central part; (**b**) detail.

The microstructure after quenching consists of low-carbon martensite and a small proportion of bainite (Figure 11a,b), and after tempering it corresponds to tempered martensite (Figure 12a,b) [39].

#### 3.3.3. Surface Roughness Measurement

The Table 9 presents the results of surface roughness measurement on the three lines in the same manner as it was for the carbon steel. They are analyzed in detail in the discussion section. The horizontal parameter the mean width *RSm* shows such a large variance of values, that it cannot be taken to any trustworthy consideration.

## 4. Discussion

A set of 36 triplets of surface roughness parameters, which were assigned to the respective values of four different material characteristics (Young’s modulus of elasticity, hardness, ultimate tensile strength and ductility), was examined, in search of some dependency or correlation. The observed findings will be described and their possible connection with the AWJ operation considered.

The first finding was obvious and undisputable: the range of changes in *Ra* (in the same line position) varied from 41% to 54% for the same material; it is clear that such changes cannot correspond to Young’s modulus values, which are never influenced by heat treatment to such an extent This finding corresponds to a discrepancy between the modulus values and respective mean declination angles referred to in [26]. It can, therefore, reasonably be stated that the modulus is not significantly responsible for the surface roughness.

As for other mechanic characteristics, all measured values were studied and compared, and various dependencies were analyzed. No obvious conclusions were discovered, although it is evident that there should be some correlation. The mechanism of the AWJ operation is inherently different from that of solid tools, and so macroscopic mechanical properties may exhibit contradictory effects from the point of view of AWJ machinability. The steel microstructure can then represent the decisive factor for a final classification. For example, a hard material may induce particle breakage followed by a decrease of jet efficiency, but at the same time it should prevent the abrasives from making scratches in the material and should result in a lower roughness of the cut if the material microstructure is homogenous.

The abrasive waterjet operation can be classified as a form of impact abrasive machining; it uses a mixture of fluid and solid particles, impacts the surface of a target workpiece and causes either permanent deformation or material removal [40]. In our AWJ machine, the abrasive mass flow rate is 32.5 g/s and the nominal size of particles is 0.18 mm; this amounts to ~4 × 10^6^ particles per second, which is only four particles per microsecond. At an impact velocity of several hundreds of m/s, the full process of particle indentation and rebound lasts a fraction of µs [40]. Considering that the jet spreads over a ~1 mm spot size, i.e., ~40× the area for a single particle impact, we realize that it is reasonable to treat AWJ as a sequence of single particle impacts: each particle indents, removes material and rebounds away from the target as an independent event without interference from other particles or from workpiece motion [40]. The material response to the impact is described either as ductile or brittle. Generally, ductile erosion is relevant to metals and similar materials that are capable of significant plastic deformation. Brittle erosion applies to materials that crack and fragment under impact. However, from a microscopic point of view, the type of erosion is to a great extent influenced by the microstructure of the material, namely by the fact that it is either homogeneous or that it is a mixture of several components with different characteristics [40].

In the design of our experiment, it was expected that the roughness characteristic that might trace the individual particle impact on the material would be the horizontal one: the mean width *RSm*. This expectation, however, appeared to be false: the changes of *RSm* did not prove any correlation to either the mechanical characteristics or the visible microstructure dimensions. Moreover, it is evident that the values of *RSm* at the bottom lines are burdened with large uncertainty. When material is cut with a traverse speed that ensures just a medium quality, as was the case in our experiments, the bottom lines show considerable waviness rising from both pressure fluctuations and a decrease of jet energy along the sample profile. This leads to a distortion of *RSm* values that is so extensive that on the bottom lines they do not have any informative value.

Having compared the results of the vertical roughness parameters of all three grades of steel, some interesting findings appeared. *Ra* reached its maximum value after pure quenching for two steels: carbon steel C45 and low-alloy steel 30CrV9 (Figure 3 and Figure 11, Table 5 and Table 9). The manganese-silicon steel exhibited a different behavior: the surface roughness dropped down significantly after pure quenching, although both the hardness and ultimate tensile strengths increased (Figure 7, Table 7). The quenched and tempered 37MnSi5 sample was cut with the lowest surface roughness (Figure 8, Table 7).

The changes in *Rz* are similar, with the range of changes being just a bit lower. This result could be anticipated; it corresponded to the fact that the samples were handled with care so that no additional scratches or damage were generated. Although *Rz* is preferred to *Ra* by the contact tester manufacturer, in the case of normal AWJ cutting *Ra* is usually more important.

**Figure 13:**
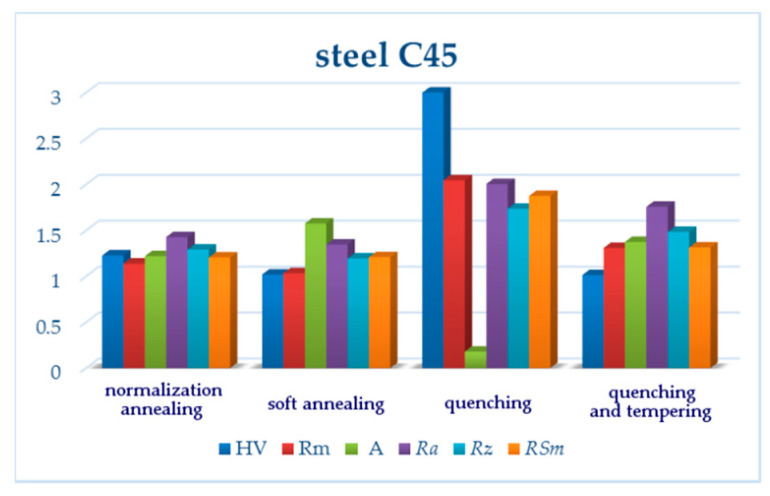
Relative changes of mechanical characteristics and measured surface roughness parameters for C45. All values were normalized, being divided by respective reference values (150 HV, 560 MPa, 16%—values for raw C45 steel [37]; *Ra*, *Rz, RSm* values for the upper line of the normalization annealing sample).

Another interesting finding seems to be the difference of the roughness results for the carbon steel and the two remaining ones. The quenched and tempered carbon steel had worse surface parameters than both types of annealed carbon steel (Table 5); in the case of the steels with lower contents of carbon, it was the other way around (Table 7 and Table 9). The Vickers hardness HV30 measured on carbon steel was lower, but the ultimate tensile strength was higher (Table 4, Figure 13). The reason for the worse AWJ machining may, therefore, rather correlate with the steel microstructure. The steel microstructure analysis revealed that the final microstructure of the carbon steel was formed mainly by bainite with a ferritic network along the grain boundaries (Figure 4). This microstructure is likely to contain more internal stresses [41], which tend to release during the cutting and cause a higher surface roughness after AWJ cutting.

Quenched and high temperature tempered 37MnSi5 and 30CrV9 were both martensitic with a low proportion of bainite and ferrite (Figure 8 and Figure 12); however, their elemental composition is different, and this leads to a different way of precipitating carbon. Both manganese and silicon tend to deposit in the solid phase and strengthen it. In contrast, both chromium and vanadium are elements that induce carbide formation in the material; therefore, the carbides are present in this steel grade, although the carbon content is lower than for manganese steel.

**Figure 14:**
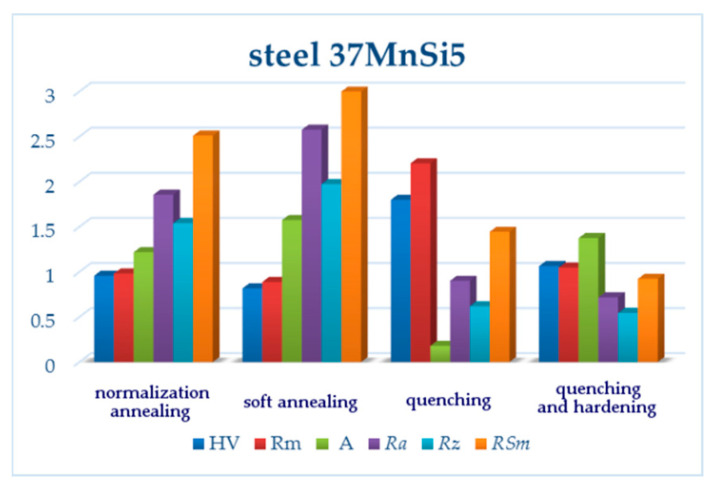
Relative changes of mechanical characteristics and measured surface roughness parameters for 37MnSi5. All values were normalized, being divided by respective reference values (170 HV, 880 MPa, 19%; *Ra*, *Rz, RSm* values for the upper line of the normalization annealing sample).

**Figure 15:**
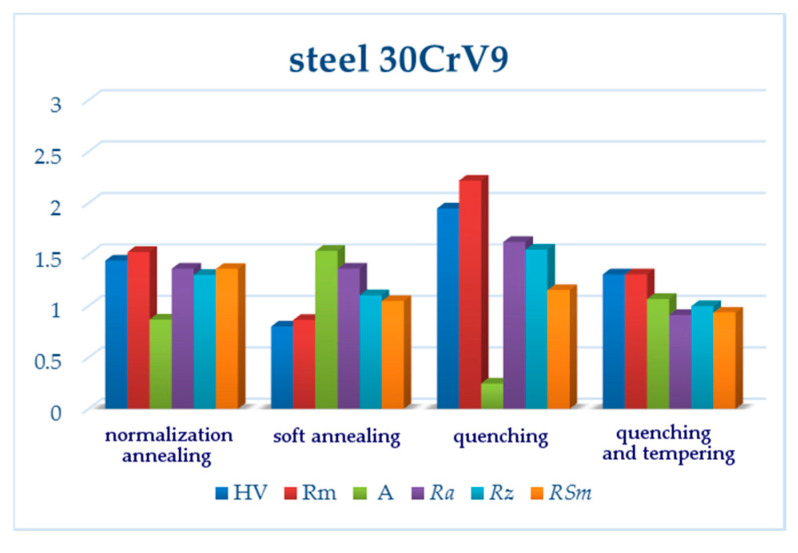
Relative changes of mechanical characteristics and measured surface roughness parameters for 30CrV9. All values were normalized, being divided by respective reference values (150 HV, 790 MPa, 15%; *Ra*, *Rz, RSm* values for the upper line of the normalization annealing sample).

Both normalization and soft annealing led to a decrease of both the hardness and ultimate tensile strength; therefore, it is not surprising that *Ra* and *Rz* decreased as well (Figure 14 and Figure 15). The microstructure of individual steel grades was identified in comparison with similar grades published in the literature, such as [42].

The abovementioned findings can be summarized, specifying the individual behavior of the examined steel grades subjected to four types of heat treatment:

(1) C45 steel exhibits the effect of a higher carbon content, which increases the hardness and yield strength R_m_. The roughness values also increase for this steel grade, due to the presence of different structural components; the inhomogeneous microstructure after normalization annealing consists of ferrite (F)—soft phase and perlite (P)—harder phase. After hardening, there are three structural components with different hardness in the microstructure: martensite (M)—the hardest, P—softer, F—the softest. Two main phases (tempered M and B, softer than M but harder than P) appear again after hardening and tempering.

(2) 37MnSi5 steel contains less carbon; moreover, the presence of Mn and Si strengthen the solid solution. The roughness values are lower than for C45 steel. After normalization and soft annealing, two structural components occur in the microstructure: ferrite and perlite; meanwhile, after quenching and quenching and tempering, the microstructure is quite homogeneous, formed by M and B with a small proportion of ferrite, and therefore the roughness values are low when compared to those of carbon steel, although the strength limits are relatively higher than for C45 steel.

(3) Although 30CrV9 steel has a low carbon content (see Table 1), the roughness values are relatively high, probably due to the content of Cr and V-based carbides, which precipitate the matrix in the state after hardening and hardening and tempering. In the normalized state and the state after soft annealing, the influence of the inhomogeneous microstructure is again manifested, which will have an effect on the impact of abrasive particles on the individual structural components, and thus on the increase of the roughness value.

## 5. Conclusions

This paper presents findings of a new research topic that represents an interdisciplinary theme requiring the cooperation of researchers from several fields. They can be summarized as follows:

1. Young’s modulus is not significantly responsible for the surface roughness of steel materials cut with an abrasive water jet.
2. Pure quenching lowers the roughness of the AWJ cut if it is accompanied with the substantial growth of the ultimate tensile strength and homogeneous steel microstructure.
3. The carbon contents in steel appear to be the primary factor influencing the behavior of the steel grade in terms of AWJ machinability.
4. The homogeneity of the steel microstructure is another important factor. The higher the difference in hardness of structural components (martensite, bainite, lamellar pearlite, spheroidized pearlite, ferrite) in the inhomogeneous microstructure, the higher the roughness values.

In our future research, these findings will be verified on other steel grades, and the correlation between the surface roughness parameters and the declination angle will be studied. Measurements with various traverse speeds and sample thicknesses will enable us to study in detail the mechanism of AWJ interaction with material and to derive cutting parameter optimization proposals for heat-treated steels.

## Figures and Tables

**Table 1:** Chemical composition of examined steels in wt. %.

Steel Grade	C	Mn	Si	P	S	Cr	Ni	Mo	V
C45	0.42	0.66	0.29	0.02	0.01	0.08	0.05	0.01	0.00
37MnSi5	0.39	1.18	1.27	0.03	0.01	0.08	0.04	0.01	0.02
30CrV9	0.27	0.53	0.29	0.02	0.02	2.85	0.05	0.01	0.17

**Table 2:** The heat treatment: recommended and real (last column) values.

Heat Treatment	C45	37MnSi5	30CrV9	Real Value
normalization annealing	840–870/air	850–890/air	860–900/air	865 ^1^/air
soft annealing	680–720/furnace	680–720/furnace	700–740/furnace	710 ^1^/furnace
quenching	800–830/water	820–850/water	830–880/water	830 ^1^/water
quenching and tempering	530–670/water, air	530–680/water, air	550–660/water, air	600 ^1^/ air

^1^ The temperature within all intervals was chosen.

**Table 3:** AWJ cutting parameters.

Cutting Parameter [unit]	Value
working pressure [MPa]	380
water flow rate [L/min]	1.9
water nozzle diameter [mm]	0.25
abrasive tube diameter [mm]	1.02
type of abrasive	Australian garnet
mesh	80 ^1^
abrasive mass flow rate [g/min]	225
stand-off distance [mm]	2
traverse speed [mm/min]	100

^1^ Mean diameter of particles should be approximately 180 μm.

**Table 4:** Vickers hardness HV30, lower R_eL_ and upper R_eH_ yield strength, ultimate tensile strength R_m_, percentage elongation after fracture A.

Steel C45	HV30	R_eL_ [MPa]	R_eH_ [MPa]	R_m_ [MPa]	A [%]
normalization annealing	184 ± 1	372.5 ± 2.5	381.8 ± 2.3	636.5 ± 2.9	19.5 ± 4.8
soft annealing	153 ± 4	274.1 ± 1.4	278.9 ± 2.4	577.9 ± 0.4	25.2 ± 2.8
quenching	458 ± 14	739.5 ± 9.8 ^1^	- ^1^	1145.2 ± 7.8	2.87 ± 1.9
quenching and tempering	152 ± 2	492.6 ± 22.1	506.5 ± 24.7	732.7 ± 13.3	22.1 ± 1.6

^1^ The proof strength R_p0.2_ had to be evaluated for quenched material.

**Table 5:** Surface roughness characteristics after abrasive waterjet cutting.

Roughness Parameter		Normalization Annealing	Soft Annealing	Quenching	Quenching and Tempering
*Ra*	upper	7.69 ± 1.12	7.67 ± 0.81	9.56 ± 0.82	8.38 ± 1.21
	central	10.97 ± 1.05	10.35 ± 1.53	15.42 ± 1.88	13.51 ± 1.66
	bottom	21.04 ± 2.25	16.58 ± 2.89	22.20 ± 2.52	20.65 ± 4.88
*Rz*	upper	43.72 ± 4.23	44.17 ± 3.78	50.42 ± 5.28	46.70 ± 4.57
	central	56.43 ± 6.86	52.22 ± 7.63	75.87 ± 10.66	64.78 ± 6.22
	bottom	97.03 ± 12.07	80.57 ± 7.63	102.17 ± 11.10	96.43 ± 20.31
*RSm*	upper	713.33 ± 113.16	723.85 ± 142.80	1025.58 ± 186.18	748.88 ± 192.38
	central	860.18 ± 79.28	862.72 ± 134.19	1338.57 ± 196.84	937.60 ± 81.18
	bottom	1249.15 ± 142.80	1056.90 ± 220.03	1165.18 ± 115.64	1054.15 ± 218.15

**Table 6:** Vickers hardness HV30, lower R_eL_ and upper R_eH_ yield strength, ultimate tensile strength R_m_, percentage elongation after fracture A.

Steel 37MnSi5	HV30	R_eL_ [MPa]	R_eH_ [MPa]	R_m_ [MPa]	A [%]
normalization annealing	163 ± 2	546.1 ± 40	562.5 ± 38.7	864.3 ± 43.8	22.6 ± 1.3
soft annealing	139 ± 2	495.6 ± 3.8	499.2 ± 5.2	782.0 ± 6.3	27.5 ± 2.6
quenching	306 ± 8	912.0 ± 29.5 ^1^	- ^1^	1940.4 ± 21.7	3.86 ± 1.5
quenching and tempering	181 ± 1	724.4 ± 12.4	-	922.1 ± 8.0	19.8 ± 0.3

^1^ The proof strength R_p0.2_ was evaluated for hardened material.

**Table 7:** Surface roughness characteristics after abrasive waterjet cutting.

Roughness Parameter	Normalization Annealing	Soft Annealing	Quenching	Quenching and Tempering
	3.57 ± 0.32	3.63 ± 0.30	1.86 ± 0.14	2.00 ± 0.22
*Ra*	6.63 ± 0.63	9.20 ± 1.45	3.22 ± 0.45	2.56 ± 0.24
	17.39 ± 3.27	21.41 ± 2.65	5.56 ± 0.82	3.61 ± 0.45
	25.13 ± 2.16	24.50 ± 6.78	10.92 ± 1.13	13.68 ± 1.04
*Rz*	38.70 ± 4.22	49.57 ± 7.28	15.57 ± 1.45	13.68 ± 1.04
	84.13 ± 15.23	99.63 ± 11.50	23.18 ± 2.31	17.47 ± 1.83
	262.72 ± 30.01	239.73 ± 33.98	182.68 ± 30.49	148.03 ± 29.96
*RSm*	659.73 ± 98.06	791.65 ± 95.27	379.53 ± 69.09	242.63 ± 20.46
	951.48 ± 142.95	1131.05 ± 100.84	1054.53 ± 276.95	851.50 ± 367.08

**Table 8:** Vickers hardness HV30, proof strength R_p0.2_ [MPa], ultimate tensile strength R_m_ [MPa], percentage elongation after fracture A [%].

Steel 30CrV9	HV30	R_p0.2_ [MPa]	R_m_ [MPa]	A [%]
normalization annealing	216 ± 0	745.9 ± 6.8	1203.1 ± 0.2	13.3 ± 1.2
soft annealing	120 ± 2	430.5 ± 5.3	682.0 ± 5.0	22.7 ± 1.2
quenching	292 ± 8	957.3 ± 80.1	1751.7 ± 48.3	3.7 ± 0.8
quenching and tempering	196 ± 1	920.3 ± 9.1	1032.9 ± 3.4	16.2 ± 0.4

**Table 9:** Surface roughness characteristics after abrasive waterjet cutting.

Roughness Parameter	Normalization Annealing	Soft Annealing	Quenching	Quenching and Tempering
	10.03 ± 1.24	8.10 ± 1.42	10.73 ± 1.24	7.17 ± 0.93
*Ra*	13.65 ± 1.63	11.08 ± 1.70	16.26 ± 1.38	9.15 ± 1.13
	31.51 ± 2.27	26.47 ± 3.63	42.04 ± 3.75	17.60 ± 2.69
	51.27 ± 6.49	46.60 ± 6.77	56.67 ± 5.38	40.95 ± 3.66
*Rz*	66.72 ± 5.42	56.60 ± 5.74	79.32 ± 6.63	51.08 ± 8.08
	174.60 ± 13.61	144.48 ± 34.30	230.38 ± 25.25	82.50 ± 11.98
	967.97 ± 182.43	697.45 ± 142.47	995.27 ± 220.03	765.17 ± 121.69
*RSm*	1315.48 ± 131.10	1012.73 ± 185.23	1116.10 ± 112.33	904.85 ± 138.45
	2025.05 ± 292.21	2125.05 ± 340.08	1985.23 ± 172.60	1121.83 ± 140.32
